# Rearrangement of chromosome bands 12q14~15 causing *HMGA2-SOX5* gene fusion and *HMGA2* expression in extraskeletal osteochondroma

**DOI:** 10.3892/or.2015.4035

**Published:** 2015-06-05

**Authors:** IOANNIS PANAGOPOULOS, BODIL BJERKEHAGEN, LUDMILA GORUNOVA, INGEBORG TAKSDAL, SVERRE HEIM

**Affiliations:** 1 Department of Section for Cancer Cytogenetics, Institute for Cancer Genetics and Informatics, The Norwegian Radium Hospital, Oslo University Hospital, Oslo, Norway; 2 Department of Centre for Cancer Biomedicine, Faculty of Medicine, University of Oslo, Oslo, Norway; 3 Department of Pathology, The Norwegian Radium Hospital, Oslo University Hospital, Oslo, Norway; 4 Department of Radiology, The Norwegian Radium Hospital, Oslo University Hospital, Oslo, Norway; 5 Department of Faculty of Medicine, University of Oslo, Oslo, Norway

**Keywords:** chromosome bands 12q14~15, *HMGA2-SOX5* gene fusion, extraskeletal osteochondroma, *HMGA2* gene, cytogenetics, HMGA2 expression

## Abstract

We describe two cases of extraskeletal osteochon-droma in which chromosome bands 12q14~15 were visibly rearranged through a pericentric inv(12). Molecular analysis of the first tumor showed that both transcript 1 (NM_003483) and transcript 2 (NM_003484) of *HMGA2* were expressed. In the second tumor, the inv(12) detected by karyotyping had resulted in an *HMGA2-SOX5* fusion transcript in which exons 1–3 of *HMGA2* were fused with a sequence from intron 1 of *SOX5*. The observed pattern is similar to rearrangements of *HMGA2* found in several other benign mesenchymal tumors, i.e., disruption of the *HMGA2* locus leaves intact exons 1–3 which encode the AT-hook domains and separates them from the 3′-terminal part of the gene. Our data therefore show that a subset of soft tissue osteochondromas shares pathogenetic involvement of *HMGA2* with lipomas, leiomyomas and other benign connective tissue neoplasms.

## Introduction

Osteochondromas are benign neoplasms that can be subdivided into 2 groups. The more common tumors (also called osteocartilaginous exostoses) consist of a cartilage-capped bony projection arising on the external surface of bone and containing a marrow cavity that is continuous with that of the underlying bone (1). The second variant is the rare extraskeletal (or soft tissue) osteochondroma. The latter is defined as a single mass that is peripherally encased by mature hyaline cartilage, has an osseous centre, shows an overall organization similar to that of a conventional osteochondroma but the lesion cannot be intra-articular or in any way be attached to bone (2–4).

Many osteochondromas in bone are asymptomatic and consequently are not detected and resected (1). Candidates for the cell of origin of osteochondroma include growth-plate chondrocytes, perichondrial cells, and cells of the Groove of Ranvier (5,6). The latter is a fibrochondrosseous structure encircling the growth plate and containing chondro- and osteoprogenitor cells. It was long debated whether osteochon-droma was a developmental disorder or a true neoplasm. The finding in a subset of osteochondromas of cytogenetic aberrations, mainly 8q deletions, as well as biallelic inactivation of the *EXT1* (located in 8q24) or *EXT2* (located in 11p11) gene in cells of the cartilage cap supports a neoplastic nature of the tumors (1). Evidence has been provided that *EXT1* functions as a typical tumor suppressor gene, that is, both copies are functionally inactivated in osteochondroma cells, with both inactivating events being somatic in solitary, non-hereditary osteochondromas (7,8). The protein sequences EXT1 and EXT2 show structural similarities. They accumulate in the Golgi apparatus where they catalyze the synthesis of heparan sulphate (9,10), an essential component of cell surface and matrix-associated proteoglycans (11). It interacts with numerous signaling proteins and regulates their distribution and activity on target cells. Many of these proteins are expressed in the growth plate of developing skeletal elements, and several skeletal phenotypes are caused by mutations affecting these proteins as well as in heparan sulphate-synthesizing and modifying enzymes (11). Mutations of *EXT1* and *EXT2* lead to heparan sulphate deficiency. The resulting aberrant distribution of signaling factors as well as aberrant responsiveness to them by target cells lead to exostosis formation (11).

Extraskeletal osteochondroma is a slowly growing, often painless tumor which is most commonly located in the hands, feet, and knee joint (2,3,12–15). Extraskeletal osteochondromas have also been found near the hip (16), in the buttocks (17), and in the nape area (18). They may display cellular atypia but they do not metastasize or undergo malignant transformation (2–4). Although various mechanisms have been suggested for the cause of extraskeletal osteochondroma (3,4), its etiology and pathogenesis are unknown and there is not cytogenetic or molecular genetic information about the disease.

In the present study, we describe the cytogenetic rearrangement of chromosome band(s) 12q14~15 through a pericentric inversion in two extraskeletal osteochondromas. The molecular result of this was aberrant expression of *HMGA2*.

## Materials and methods

### Ethics statement

The study was approved by the Regional Ethics Committee (Regional komité for medisinsk forskning-setikk Sør-Øst, Norge, http://helseforskning.etikkom.no) and written informed consent was obtained from the patients.

### Patients

**Figure 1:**
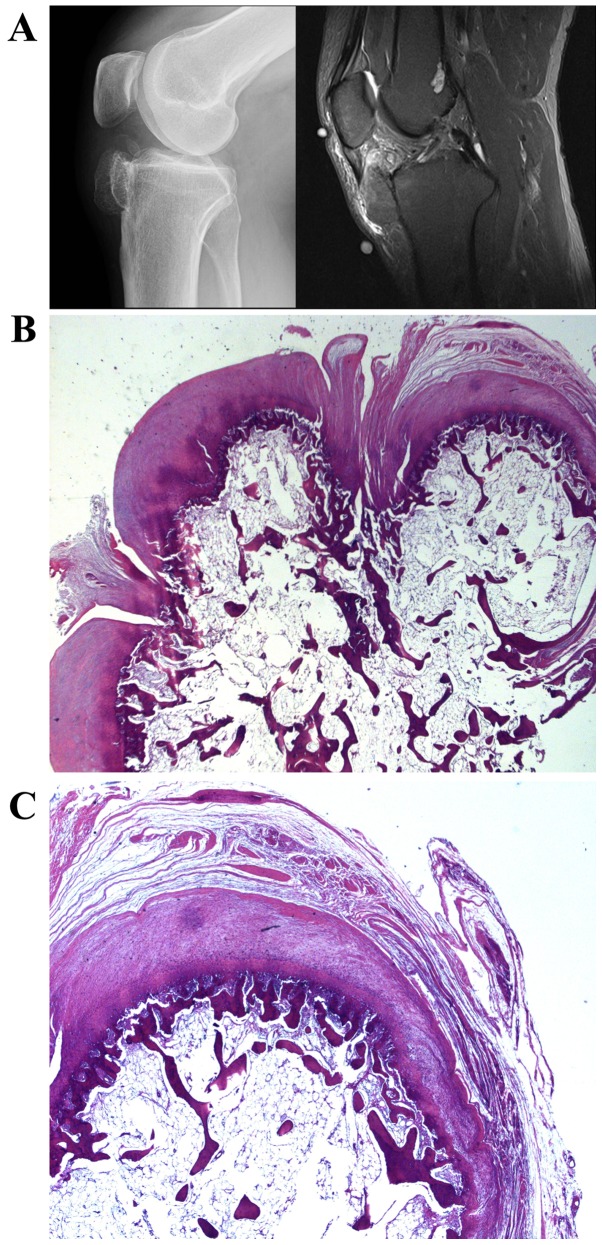
Case 1. Extraskeletal osteochondroma. (A) Lateral radiograph and MR sagittal STIR image (incidental high-signal lesion in the femur has no relevance). (B and C) H&E-sections showing the osteochondromatous tumor in the knee centrally consisting of fatty marrow and a cartilage cap.

Case 1. A 43-year-old man had noticed a tumor in the right knee for the last five years. For six months there had been some pain. The plain radiograph showed a bony lesion adjacent to the proximal tibial epiphysis and metaphysis. It was ovoid to dumbbell-shaped, measured 4 cm, and consisted of trabecular bone surrounded by a thin cortex. The MR signal was consistent with fatty bone marrow covered by a thin cartilage cap. Thus, the lesion resembled an osteochondroma, but the underlying anterior tibial cortex was intact and there was no continuous marrow cavity with the underlying bone (Fig. 1A). Histological examination disclosed a bony lesion in which fatty medullary bone in the middle was surrounded by thin cortical bone covered by a cartilaginous cap. Around the cap there was some fibrocartilage and connective tissue. No cellular atypia was observed. The microscopic features were of an osteochondromatous lesion with no connection to preexisting bone (Fig. 1B and C).

**Figure 2:**
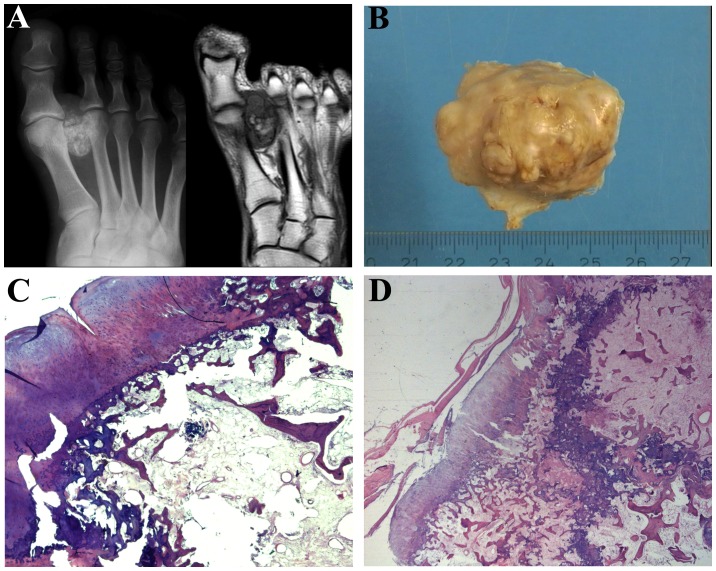
Case 2. Extraskeletal osteochondroma. (A) Radiograph and T1-weighted MR image. (B) Gross specimen. (C and D) H&E-sections showing the osteochondromatous tumor in the foot consisting of fatty marrow with sclerotic bone and a cartilage cap.

Case 2. A 45-year-old man had for at least five years noticed a growing tumor in the foot. The radiograph revealed a partly mineralized 4-cm lesion in the soft tissues between the first and second metatarsal heads and extending distal to the MCP-joints. There was no continuous cortex-like periphery, and the calcifications were partly flocculent and comma-shaped as in cartilage matrix, partly more trabecular resembling ossification. On MR, the lesion was located adjacent to the surface of the first metatarsal and the base of the proximal phalanx without involvement of the bone marrow. There was fatty marrow in the central part, while the periphery of the lesion gave signals as fibrous and chondroid tissue (Fig. 2A). The excised tumor consisted of fatty and fibrous bone marrow centrally with small trabeculae and some more sclerotic bony tissue without atypia (Fig. 2B). On the surface a cartilage cap was noted, but there was no continuum of the medullary with the underlying bone marrow. The microscopic features were of an osteochondromatous lesion with no connection to the pre-existing bone (Fig. 2C and D).

### Chromosome banding analysis and fluorescence in situ hybridization (FISH)

Samples from the surgically removed tumors were mechanically and enzymatically disaggregated and short-term cultured as described elsewhere (19). The cultures were harvested and the chromosomes G-banded using Wright stain. The subsequent cytogenetic analysis and karyotype description followed the recommendations of the ISCN (20).

FISH analysis was performed on metaphase plates. BAC clones were retrieved from the Human genome high-resolution BAC re-arrayed clone set (the ‘32K set’; BACPAC Resources, http://bacpac.chori.org/pHumanMinSet.htm). The ‘32K set’ is mapped on the UCSC Genome Browser on Human Genome May 2004 (NCBI/hg17) assembly. Mapping data for the 32K human re-array are available in an interactive web format (http://bacpac.chori.org/pHumanMinSet.htm, from the Genomic Rearrays page) and are obtained by activation of the UCSC browser track for the hg17 UCSC assembly from the ‘32K set’ homepage (http://bacpac.chori.org/genomicRear-rays.php). The BAC clones were selected according to physical and genetic mapping data on chromosome 12 as reported on the Human Genome Browser at the University of California, Santa Cruz website (May 2004, http://genome.ucsc.edu/). In addition, FISH mapping of the clones on normal controls was performed to confirm their chromosomal location.

**Figure 3:**
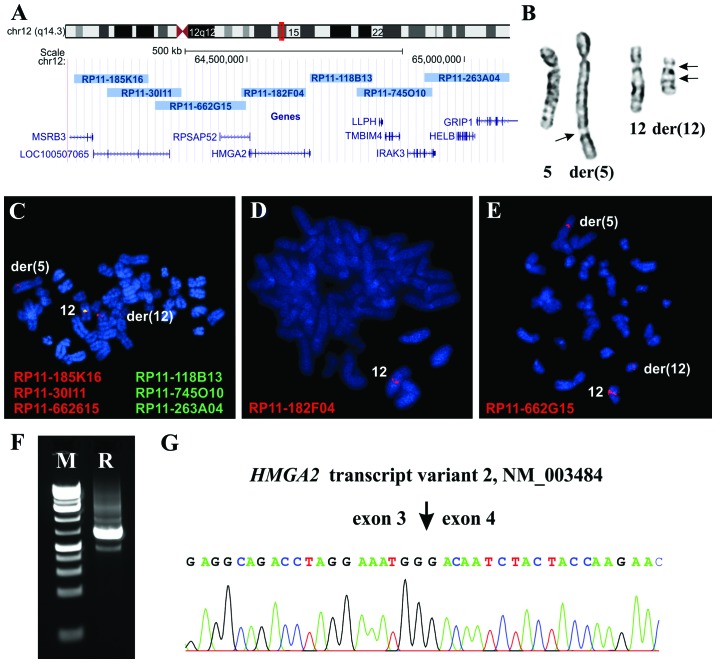
Extraskeletal osteochondroma with expression of *HMGA2* transcripts 1 and 2. Cytogenetic, FISH and PCR analyses of case 1. (A) Chromosome 12 ideogram with the location of the *HMGA2* locus and the location of the BACs used for FISH experiments. The investigated region is shown as a red box on the chromosome 12 ideogram. (B) Partial karyotype showing the chromosome aberrations der(5)t(5;12)(q35;q14~15) and der(12)t(5;12)inv(12)(p11q14~15) together with the corresponding normal chromosomes. Arrows indicate breakpoints. (C) FISH showing that the green signal (pool of the BACs RP118B13, RP11-745O10, and RP11-263A04) is deleted and that the red signal (pool of the BACs RP11-185K16, RP11-30I11, and RP11-662G15) is split and seen on both der(5) and der(12) chromosomes. (D) FISH showing that the probe RP11-182F04 is seen as one copy on normal chromosome 12. (E) FISH showing that the RP11-662G15 is split and seen on der(5), der(12), and normal chromosome 12. (F) 3′-RACE amplified a single cDNA fragment (lane R). (G) Partial sequence chromatogram of the 3′-RACE amplified cDNA fragment showing (arrow) the junction of exon 3 and exon 4 of *HMGA2* transcript variant 2 with accession number NM_003484. FISH, fluorescence in situ hybridization.

The clones used were RP11-185K16, (chr12:64103524-642 74514), RP11-30I11 (chr12:64178505-64349708), RP11-662 G15 (chr12:64288763-64498219), RP11-182F04 (chr12:644 86880-64635771), RP118B13 (chr12:64644 968-64789255), RP11-745O10 (chr12:64752327-64926193) and RP11-263A04 (chr12:64908453-65103538). All of them are mapped to chromosome band 12q14.3 (Fig. 3A). DNA was extracted and probes were labeled and hybridized according to Abbott Molecular recommendations (http://www.abbottmolecular.com/home.html). Chromosome preparations were counter-stained with 0.2 *µ*g/ml DAPI and overlaid with a 24×50 mm^2^ coverslip. Fluorescent signals were captured and analyzed using the CytoVision system (Leica Biosystems, Newcastle, UK).

### Molecular genetic analysis

Total RNA was extracted using miRNeasy kit and QIAcube according to the manufacturer’s recommendations (both from Qiagen Nordic, Stockholm, Sweden). Human Universal Reference Total RNA was used as control (Clontech Laboratories; Takara-Bio Group; Europe/SAS, Saint-Germain-en-Laye, France). According to the company’s information, it is a mixture of total RNAs from a collection of adult human tissues chosen to represent a broad range of expressed genes. Both male and female donors are represented. Total RNA (400–500 ng) was reverse-transcribed in a 20-*µ*l reaction volume using iScript Advanced cDNA Synthesis kit for RT-qPCR according to the manufacturer’s instructions (Bio-Rad Laboratories, Oslo, Norway). The cDNA was diluted to 10 ng equivalent of RNA/*µ*l and 1 *µ*l was used as template in subsequent real-time PCR assays.

Real-time PCR was carried out to determine the expression level of *HMGA2*. The TaqMan gene expression assays (Applied Biosystems, Foster City, CA, USA), Hs00171569_m1 (HMGA2 exons 1–2), Hs00971725_m1 (HMGA2 exons 4–5), Hs00609162_m1 (EXT1 exon 8–9), and Hs00181158_m1 (EXT2 exon 8–9) were used. The *S100A10* gene, assay Hs00237010_ml, was used as endogenous control since this gene is expressed in chondrocytes (21). The 20-*µ*l reaction volume contained 1× TaqMan Universal Master Mix II with UNG, lx of the 20X TaqMan gene expression mix and 1 *µ*l cDNA (10 ng equivalent of RNA). Four replicates of each sample and the endogenous control were performed. Real time PCR was run on a CFX96 Touch™ Real-Time PCR Detection system (Bio-Rad). The thermal cycling included an initial step at 50°C for 2 min, followed by 10 min at 95°C and 40 cycles of 15 sec at 95°C, and 1 min at 60°C. The data were analyzed using the CFX Manager software (Bio-Rad).

For 3′-RACE, 100 ng of total RNA were reverse-transcribed in a 20-*µ*l reaction volume with the A3RNV-RACE primer (5′-ATC GTT GAG ACT CGT ACC AGC AGA GTC ACG AGA GAG ACT ACA CGG TAC TGG TTT TTT TTT TTT TTT-3′) using iScript Select cDNA Synthesis kit according to the manufacturer’s instructions (Bio-Rad). One microliter was used as template and amplified using the outer primer combination HMGA2–846F1 (5′-CCA CTT CAG CCC AGG GAC AAC CT-3′) and A3R-1New (5′-TCG TTG AGA CTC GTA CCA GCA GAG TCA C-3′). One microliter of the amplified products was used as template in nested PCR with the primers HMGA2–982F1 (5′-CAA GAG TCC CTC TAA AGC AGC TCA-3′) and A3R-3 (5′-CGA GAG AGA CTA CAC GGT ACT GGT-3′). For both PCRs the 25-*µ*l reaction volume contained 12.5 *µ*l of Premix Taq (Takara-Bio) template, and 0.4 *µ*M of each of the forward and reverse primers. PCR cycling consisted of an initial step of denaturation at 94°C for 30 sec followed by 35 cycles of 7 sec at 98°C, 30 sec at 55°C, 90 sec at 72°C, and a final extension for 5 min at 72°C.

Three microliters of the PCR products were stained with GelRed (Biotium, Hayward, CA, USA), analyzed by electrophoresis through 1.0% agarose gel, and photographed. The rest of the amplified fragments were purified using the Thermo Scientific GeneJET PCR purification kit (Fisher Scientific, Oslo, Norway) and direct sequencing was performed using the Lightrun sequencing service of GATC Biotech (http://www.gatc-biotech.com/en/sanger-services/lightrun-sequencing.html). The BLAST (http://blast.ncbi.nlm.nih.gov/Blast.cgi) and BLAT (http://genome.ucsc.edu/cgi-bin/hgBlat) programs were used for computer analysis of sequence data.

To verify the results obtained by 3′-RACE in case 2, i.e., the presence of an *HMGA2-SOX5* chimera transcript (see below), PCRs were performed using the following primer combinations: HMGA2-846F1/SOX5-Int1-R1 (5′-CAA CCA TAG CTG CAT CCC GCT GT-3′), HMGA2-846F1/SOX5-634R1 (5′-AAG TTC CCC GAT CCC ATT GCA AG-3′) and HMGA2-846F1/SOX5-481R1 (CGT TCA GGA GTT CCC AGG GCT GT). The primers SOX5-634R1 and SOX5-481R1 correspond to nucleotides 634–656 (exon 4) and 481–503 (exon 3) in the *SOX5* mRNA sequence with accession NM_006940 version 4. The 25-*µ*l PCR volumes contained 12.5 *µ*l of Premix Taq (Takara-Bio), 2 *µ*l of diluted cDNA, and 0.2 *µ*M of each of the forward and reverse primers. The PCRs were run on a C-1000 Thermal cycler (Bio-Rad). The PCR conditions were: an initial denaturation at 94°C for 30 sec followed by 35 cycles of 7 sec at 98°C, 120 sec at 68°C, and a final extension for 5 min at 68°C.

### Immunohistochemistry

To detect the HMGA2 protein, immu-nostaining was performed as previously described (22).

## Results

### Chromosome banding analysis and FISH

**Figure 4:**
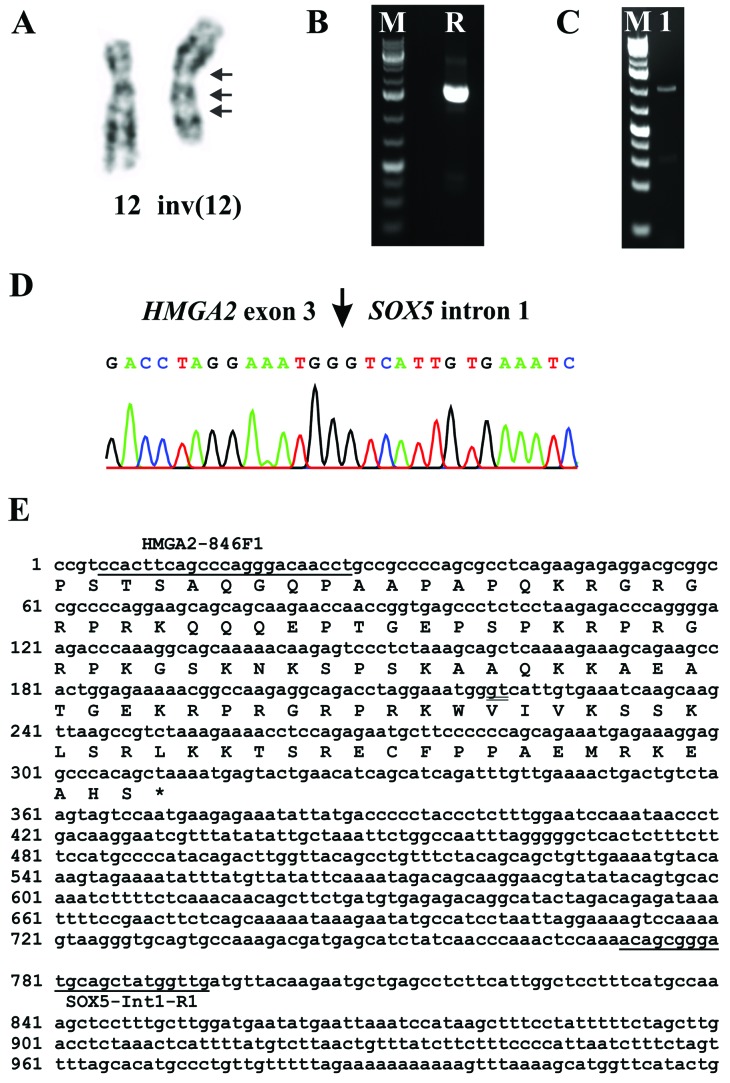
Extraskeletal osteochondroma with *HMGA2-SOX5* fusion transcript. Cytogenetic and PCR analyses of the case 2. (A) Partial karyotype showing the inv(12) (see text). (B) 3′-RACE amplified a single cDNA fragment (lane R). (C) RT-PCR amplification using the primers HMGA2-846F1/SOX5-Int1-R1 (lane 1). Lane M is the 1 kb Plus DNA ladder (GeneRuler, Fermentas). (D) Partial sequence chromatogram of the 3′-RACE amplified cDNA fragment showing (arrow) the fusion of exon 3 of *HMGA2* with a sequence from intron 1 of *SOX5*. (E) Partial sequence of the 3′-RACE-amplified cDNA fragment. The primers HMGA2-846F1 and SOX5-Int1-R1 are underlined; the junction of *HMGA2* and *SOX5* is double underlined. The putative coding sequence is shown under the nucleotide sequence. The symbol * corresponds to the ‘taa’ stop codon.

In case 1, the G-banding analysis yielded the karyotype 46,XY,der(5)t(5;12) (q35;q14~15),der(12)t(5;12)inv(12)(p11q14~15)[8]/46,XY[3] (Fig. 3B). In case 2, the analysis yielded the karyotype 46,XY,inv(12)(qter->q14~15::p11->q13::q14~15->q13::p11->pter) [13]/46,XY,idem,t(5;13)(q13;p11)[2] (Fig. 4A).

The FISH experiments in case 1 showed that there was only one copy of the *HMGA2* gene in the metaphase cells with the aberrant karyotype which was located on the normal chromosome 12 (Fig. 3C and D) indicating heterozygous deletion of *HMGA2*. On the derivative chromosomes, the probe which was a pool of the BACs RP11-118B13, RP11-745O10 and RP11-263A04 (Fig. 3C) as well as the probe RP11-182F04 which covers the *HMGA2* gene (Fig. 3D) were deleted. The probe containing the three BACs RP11-185K16, RP11-30I11 and RP11-662G15 was split with one signal on der(12) and the other on der(5) (Fig. 3C). Further experiments showed that the split signal was located on the BAC RP11-662G15 which is upstream and outside the *HMGA2* locus (Fig. 3E). Interphase FISH confirmed the heterozygous deletion of *HMGA2*. In 98 nuclei, 76 had one copy presumably corresponding to cells with an aberrant karyotype, whereas 22 had 2 copies of *HMGA2* and presumably representing cells with a normal karyotype.

### Molecular genetic analysis

3′-RACE in case 1 amplified a single fragment (Fig. 3F) which by Sanger sequencing was found to be the alternative transcript variant 2 of *HMGA2* with accession number NM_003484 (Fig. 3G).

3′-RACE in case 2 amplified a single fragment (Fig. 4B). Sanger sequencing showed that it was a chimeric cDNA fragment in which exon 3 of *HMGA2* was fused to a sequence in intron 1 of the *SOX5* gene located in 12p12 (Fig. 4D and E). PCR with the primers HMGA2-846F1/SOX5-Int1-R1 amplified a cDNA fragment (Fig. 4C), direct sequencing of which showed the same fusion point with the 3′-RACE amplified fragment (Fig. 4D and E). PCR with the forward HMGA2-846F1 primer and the reverse primers SOX5-634R1 (located in exon 4) and SOX5-481R (located in exon 3 of *SOX5*) did not amplify any other *HMGA2-SOX5* fusion transcripts.

By real-time PCR, the mean quantification cycle of *S100A10* (Cq Mean) was found to be 26.50, 22.23 and 26.75 for case 1, case 2, and the human reference control sample, respectively (Table I). The Cq Mean for *HMGA2* exons 1–2 was 34.24, 28.15 and 31.99, for case 1, case 2 and the human reference control sample, respectively. Expression of exons 4–5 of *HMGA2* was noted in case 1 and in the reference sample but not in case 2. The Cq Mean was 33.83 and 32.90 for case 2 and the reference sample, respectively. Thus, for case 2, the data indicated the presence of an *HMGA2* transcript in which exons 1 and 2 were present whereas exons 4 and 5 were lost. The expression of the *EXT1* and *EXT2* genes in case 1 was comparable to what was found in the human reference control sample whereas their expression was very low in case 2 (Table I).

### Immunohistochemistry

**Figure 5:**
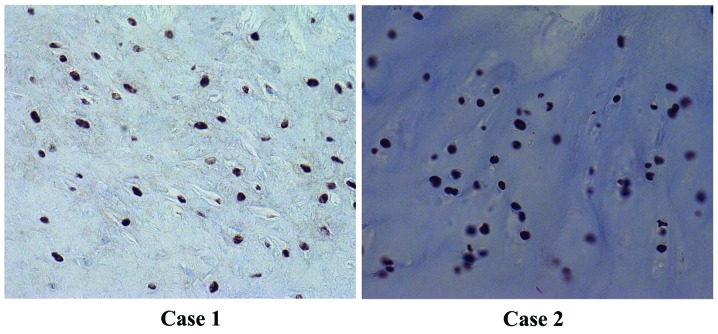
Tumor tissue showing strong and widespread immunohostochemical nuclear staining for the HMGA2 protein.

Strong and widespread immunohis-tochemical nuclear staining for HMGA2 was noted in both tumors (Fig. 5).

## Discussion

Cytogenetic information on both osteochondromas in bone and extra skeletal osteochondromas is very limited. According to the Mitelman Database of Chromosome Aberrations and Gene Fusions in Cancer (http://cgap.nci.nih.gov/Chromosomes/Mitelman, Database last updated on August 18, 2014), only 26 osteochondromas in bone with clonal karyotypic aberrations have been published in altogether 8 articles (23–30). Involvement of chromosome 8, mostly deletions of 8q as cytogenetic evidence of *EXT1* loss, was observed in 18 of them. Four other tumors showed rearrangement of 11p as cytogenetic evidence of *EXT2* involvement. Additionally, breakpoints in 1p13~22 were noted in 5 osteochondromas (29) but no gene has been associated with this cytogenetic change. No other consistent pattern of aberrations has emerged.

Here, we present two cytogenetically analyzed extraskeletal osteochondromas in which chromosome bands 12q14~15 was rearranged; both had inv(12) but no microscopically detectable rearrangement of the long arm of chromosome 8 let alone of band 8q24. Structural rearrangements involving 12q were previously reported in 7 osteochondromas (24). Aberration of 12q12~13 was found to be clonal in two tumors whereas in another two it was noted in a single metaphase only. Two other cases had abnormalities mapped to 12q24 and a third had rearrangement of 12q11 (24). It is not possible for us to know whether the breakpoint in some of these tumors might have been reassigned to 12q14~15 if reviewed again.

Since cytogenetic change of bands 12q13~15 in benign connective tissue tumors is almost always associated with rearrangement and/or activation of *HMGA2* (31), we decided to investigate whether this gene is involved also in our two cases. The experiments by 3′-RACE, RT-PCR, and immunohictochemistry showed that *HMGA2* was transcribed and translated into nuclear protein in both tumors. In case 1, the data indicated that both transcript 1 (NM_003483, real-time PCR experiments) and transcript 2 (NM_003484, 3′-RACE experiments) were expressed. *HMGA2* transcript 2 (assigned with accession numbers AF533652, AY601867, and U29112) has been found expressed in embryonic cells, cultured fibroblasts, as well as leiomyomas; evidently, expression is not restricted to neoplastic contexts (32,33).

The FISH experiments showed that, in the cells with aberrant karyotype, there was only one copy of the *HMGA2* gene which was located on the normal chromosome 12 and not on der(5) or der(12). In addition, there were cells carrying both copies of *HMGA2* and had normal 46,XY karyotype. Thus, the observed expression of *HMGA2* could be the result of an active *HMGA2* allele on the normal 12 in cells with abnormal karyo-type and/or active *HMGA2* in cells with a normal karyotype.

In case 2, only a chimeric *HMGA2-SOX5* was expressed in which exons 1–3 of *HMGA2* were fused to an intronic sequence of *SOX5* from 12p12 (Fig. 4D and E). The ensuing *HMGA2-SOX5* fusion transcript codes for a putative protein which contains amino acid residues 1–83 of HMGA2 protein (accession number NP_003474.1) corresponding to exons 1–3 of the gene, and 30 amino acid residues from the intronic sequence of *SOX5* (VIVKSSKLSRLKKTSRECFPPAEMRKEAHS). This pattern is similar to the rearrangements of *HMGA2* found in other connective tissue tumor types, i.e., disruption of the *HMGA2* locus leaves intact exons 1–3 which encode the AT-hook domains and separates them from the 3′-terminal part of the gene (34).

Recombinant HMGA2 protein was shown to significantly increase the proliferative activity of chondrocytes in a dose-dependent manner in an *in vitro* system utilizing cells of porcine origin (35). Application of a synthetic peptide comprising the functional AT-hook motifs of the HMGA2 protein onto porcine hyaline cartilage chondrocytes, grown in a monolayer cell culture, showed a growth-promoting effect similar to the wild-type HMGA2 protein (36). Moreover, *HMGA2* can influence the expression of genes involved in chondrogenesis such as *COL11A2* (37). Overexpression of *HMGA2-LPP* fusion transcripts promotes chondrogenesis by upregulating cartilage-specific collagen gene expression through the N-terminal DNA binding domains. In the same study, full-length HMGA2 was also shown to activate the *COL11A2* promoter when overexpressed indicating that *COL11A2* is a target gene of HMGA2 (37).

It should be emphasized that the role of *HMGA2* in chondromatous tumors is still studied only very rudimentary in-as-much as only six soft tissue chondromas, two skeletal chondromas, and three periosteal chondromas have been subjected to this type of analysis (38,39). *HMGA2* expression was found in four soft tissue chondromas of which three expressed a truncated transcript of *HMGA2* and one the full length transcript 1. Expression of *HMGA2* was found in both examined skeletal chondromas: a tumor with a pericentric inv(12)(p12q13) expressed a truncated transcript of *HMGA2* whereas a tumor carrying a t(2;11)(q37;q13) without visible involvement of 12q expressed the full length *HMGA2* transcript (38). On the other hand, neither conventional RT-PCR nor real-time PCR showed expression of *HMGA2* in the examined periosteal chondromas, although two of them had structural aberrations of chromosome bands 12q13~15 (39).

Our data on *EXT1* and *EXT2* from case 2 are in agreement with previously reported expression data from osteochondromas in that they showed low expression levels (7,8). For case 1, however, expression of *EXT1* and *EXT2* was at the same low level as that found in the human reference control sample (Table I). In bone osteochondromas, homozygous deletion of the *EXT1* gene is only seen in chondrocytes of the cartilaginous cap, not in cells of the perichondrium or from the bony stalk (8). A possible explanation of our results for case 1 could be that the material used for expression analysis of *EXT1* and *EXT2* contained more cells of the perichondrium and bony stalk and less cells of the cartilaginous cap.

In conclusion, our study showed that rearrangement of chromosome bands 12q14~15 is recurrent in extraskeletal osteochondromas. The cytogenetic change leads to expression of *HMGA2* or formation of *HMGA2* chimeras, i.e., the same pathogenetic motifs that are well known also from other benign connective tissue tumors.

## Figures and Tables

**Table I:** The Cq values of expression of *HMGA2*, *EXT1*, *EXT2* and *S100A10* in the examined extraskeletal osteochondromas and human reference RNA.

Gene (assay)	Case 1	Case 2	Human reference
*S100A10* (Hs00237010_ml)	26.50	22.23	26.75
*EXT1* (Hs00609162_m1)	28.47	37.93	29.07
*EXT2* (Hs00181158_m1)	28.78	32.39	27.79
*HMGA2* Exons 1–2 (Hs00171569_m1)	34.24	28.15	31.99
*HMGA2* Exons 4–5 (Hs00971725_m1)	33.83	–	32.90
